# Fibroma of the Testis and Epididymis: A Case Report on a Testis-Sparing Management Approach

**DOI:** 10.7759/cureus.116410

**Published:** 2026-09-17

**Authors:** Vladimer Papava, Valeriane Kvakhajelidze, Giorgi Sinauridze, Irakli Gakhokidze, Andro Dzneladze, Ema Gergedava, Leli Shanidze, Tamar Didbaridze, Armaz Mariamidze, Tamta Mamasakhlisi, Avtandil Imedadze, Vasil Kupradze

**Affiliations:** 1 Urology, Tbilisi State Medical University, Tbilisi, GEO; 2 Urology, Pineo Clinic, Tbilisi, GEO; 3 Radiology, Aversi Clinic, Tbilisi, GEO; 4 Radiology, Pineo Clinic, Tbilisi, GEO; 5 Urology, Lidermedi Clinic, Tbilisi, GEO; 6 Laboratory Medicine, Tbilisi State Medical University, Tbilisi, GEO; 7 Microbiology, The First University Clinic of Tbilisi State Medical University, Tbilisi, GEO; 8 Pathology, Pathology Research Center, Tbilisi, GEO; 9 Internal Medicine, Tbilisi State Medical University, Tbilisi, GEO; 10 Medicine, Tbilisi State Medical University, Tbilisi, GEO

**Keywords:** case report, epididymis, fibrous pseudotumor, magnetic resonance imaging, paratesticular fibroma, scrotal mass, testis-sparing surgery

## Abstract

Paratesticular fibromas are rare benign lesions that may clinically mimic malignancy and lead to unnecessary orchiectomy. We report the case of a 22-year-old man who presented with a firm, painless right paratesticular mass that was suspicious for neoplasm on physical examination. Serum tumor markers were normal, and imaging demonstrated multiple well-circumscribed fibrous extratesticular lesions compressing, but not invading, the testis. Surgical exploration revealed multiple firm, whitish nodules involving the epididymis and testicular tunics, and testis-sparing excision was performed. Histopathologic examination showed well-circumscribed, collagen-rich spindle-cell lesions without cytologic atypia or mitotic activity, favoring benign paratesticular fibroma. At the six-month follow-up, the patient remained asymptomatic, with no evidence of recurrence on ultrasonography. This case illustrates the potential role of organ-preserving surgery in carefully selected patients when imaging and intraoperative findings suggest a benign fibrous lesion and complete excision can be achieved without compromising oncologic safety.

## Introduction

Paratesticular fibrous lesions are uncommon benign masses arising from structures surrounding the testis, including the tunica vaginalis, tunica albuginea, epididymis, and spermatic cord. True fibromas and fibrous pseudotumors may appear similar clinically and radiologically but represent distinct pathologic processes. Fibroma of the testicular tunics is a rare benign neoplasm, whereas paratesticular fibrous pseudotumor is generally considered a reactive fibroinflammatory proliferation rather than a true neoplasm [1-3]. Both may present as firm, usually painless scrotal nodules and can mimic testicular or paratesticular malignancy [4,5].

The pathogenesis of fibrous pseudotumor remains incompletely understood. Associations with antecedent trauma, epididymo-orchitis, and hydrocele support a possible reactive inflammatory mechanism [2,5]. Increased numbers of immunoglobulin G4 (IgG4)-positive plasma cells have also been reported in some cases, suggesting a possible association with IgG4-related disease (IgG4-RD); however, this finding is not universal [5,6].

Ultrasonography is generally the initial imaging modality for evaluation of a scrotal mass, although the appearance of fibrous lesions is variable and may not reliably distinguish benign lesions from malignancy [2,4,7]. Paratesticular fibrous pseudotumors may demonstrate echogenicity similar to or slightly lower than that of the testis, sometimes with posterior acoustic shadowing and variable vascularity [8]. MRI may provide additional tissue characterization, as collagen-rich lesions commonly demonstrate low signal intensity on T2-weighted sequences [2,7-9]. Nevertheless, definitive diagnosis frequently depends on histopathologic examination.

Accurate preoperative and intraoperative assessment is important when preservation of normal testicular tissue is feasible. In patients with normal serum tumor markers and imaging findings suggestive of an extratesticular origin, local excision with intraoperative frozen-section assessment may allow testis-sparing management when the lesion can be separated from the testicular parenchyma [3,10]. We present a case of multiple fibrous lesions involving the epididymis and testicular tunics in a 22-year-old man managed with organ-preserving surgery.

## Case presentation

Clinical presentation

A 22-year-old man presented with a painless mass in the right hemiscrotum. He denied any history of preceding trauma, constitutional symptoms, urinary symptoms, or scrotal infection. Physical examination revealed a firm, irregular, non-tender mass adjacent to the epididymal region of the right testis, raising concern for a testicular or paratesticular neoplasm. Serum alpha-fetoprotein, beta-human chorionic gonadotropin, and lactate dehydrogenase levels were within normal limits. Semen analysis was also normal.

Ultrasonographic findings

Scrotal ultrasonography demonstrated a normally positioned right testis measuring 57 × 26 × 33 mm, with an estimated volume of 26.5 cm³. The testicular parenchyma was homogeneous, with normal echogenicity and preserved intratesticular vascularity. The epididymal head measured 17 × 10 mm and had normal echotexture. An 8-mm epididymal head cyst, two small hydatids measuring approximately 10 × 5 mm each, and a larger hydatid measuring 27 × 14 mm near the epididymal tail were also noted.

Although the ultrasonographic findings favored benign pathology, the firm and irregular nature of the palpable lesion remained concerning. MRI was therefore performed for further characterization.

MRI findings

MRI demonstrated multiple well-circumscribed masses closely associated with the tunica vaginalis and adjacent paratesticular tissues (Figure 1). The lesions showed low signal intensity on T2-weighted sequences, consistent with their fibrous composition (Figure 2). On contrast-enhanced fat-suppressed T1-weighted imaging, the masses demonstrated homogeneous enhancement and compressed the right testis without evidence of invasion into the testicular parenchyma, supporting an extratesticular origin (Figure 3). Overall, the MRI findings favored a benign fibrous paratesticular lesion [7,9].

**Figure 1:**
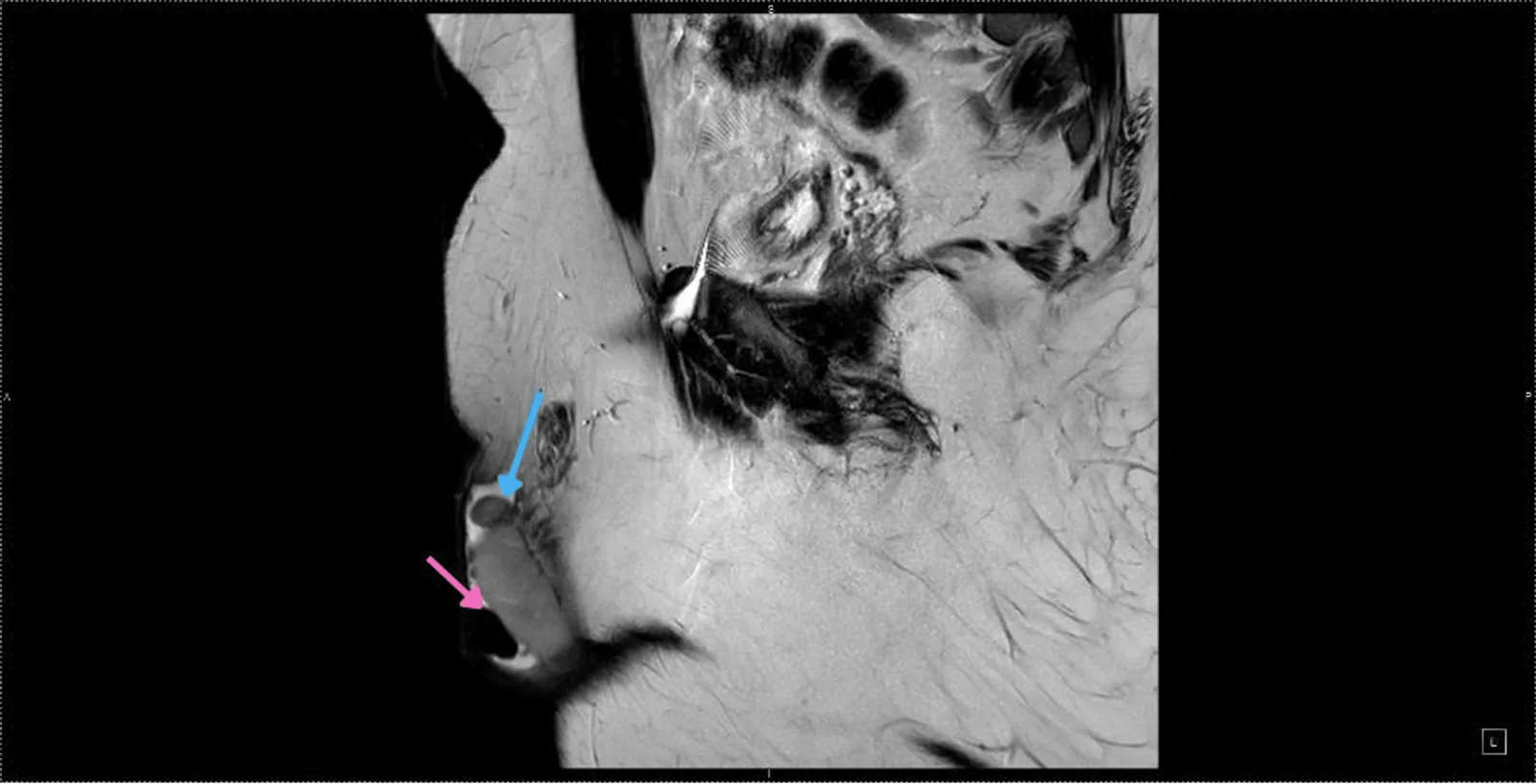
Sagittal T2-weighted image showing hypointense masses attached to the tunica vaginalis. Colored arrows indicate separate fibrous masses.

**Figure 2:**
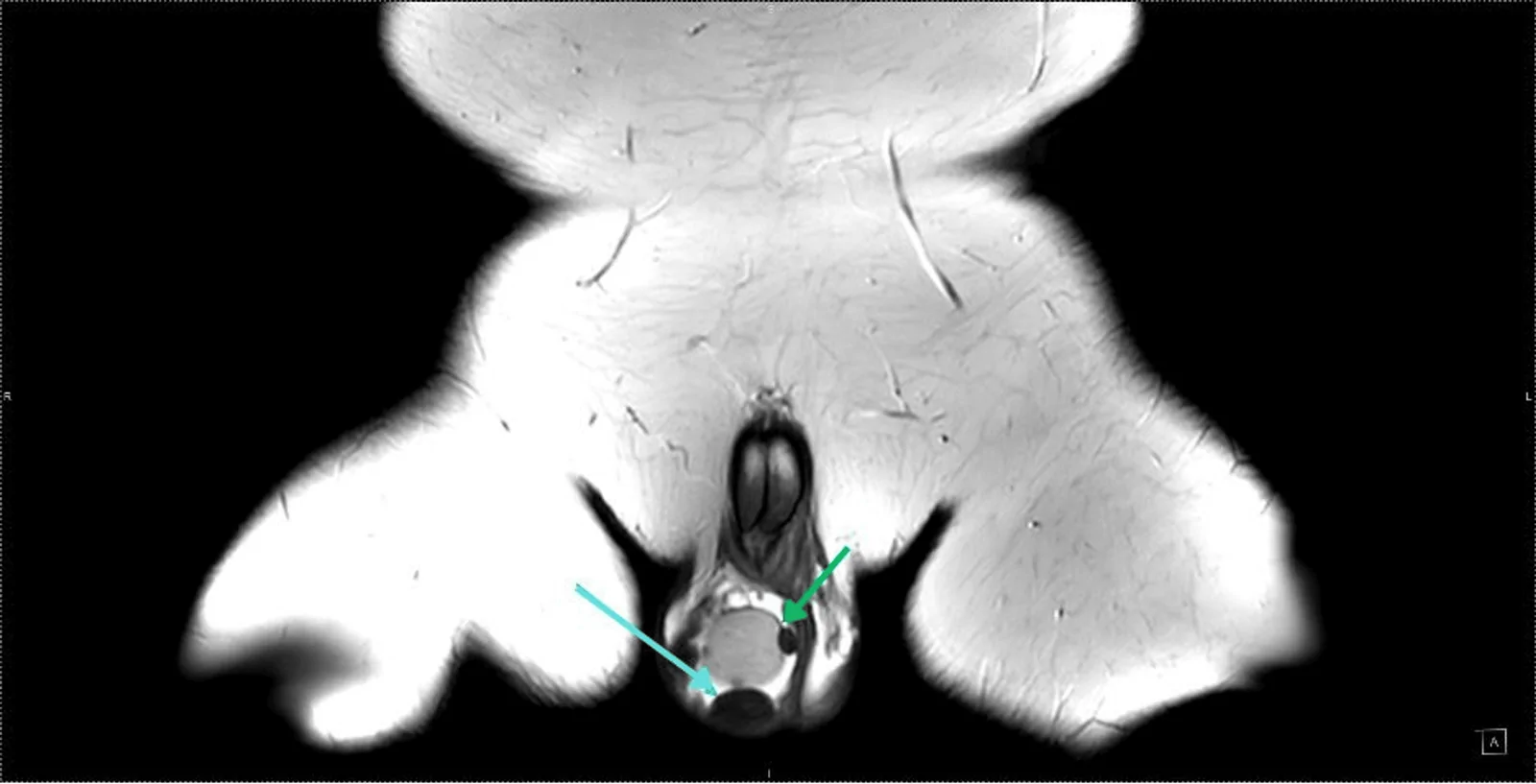
Coronal T2-weighted MRI demonstrating right extratesticular hypointense fibrous masses. Colored arrows indicate separate fibrous masses.

**Figure 3:**
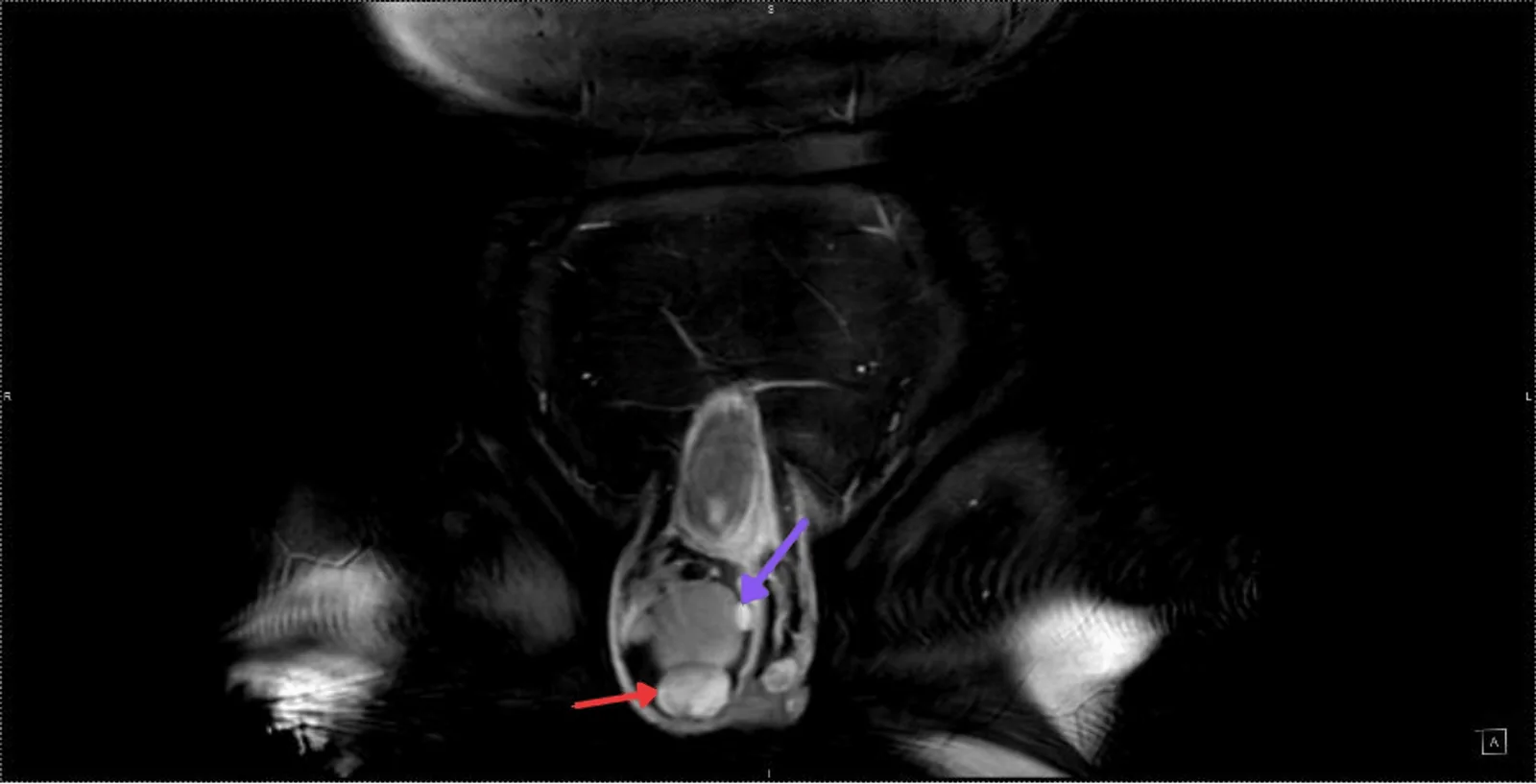
Coronal contrast-enhanced fat-suppressed T1-weighted image showing homogeneous enhancement of the extratesticular masses. Colored arrows indicate separate masses compressing the testis without invasion of the testicular parenchyma.

Surgical management

Because of the patient’s young age, the solid nature of the lesions, and the inability to exclude neoplasia with complete certainty, surgical exploration was recommended. Through a scrotal approach, multiple round, firm, whitish nodules were identified involving the epididymis and testicular tunics. Some lesions were superficially embedded within the testicular capsule, whereas others arose from the connective tissue of the epididymis.

All visible nodules were carefully excised. The involved portion of the tunica vaginalis was partially resected, and the testicular capsule was repaired with fine sutures. The testis was preserved and returned to the scrotum. The gross appearance and distribution of the lesions were considered consistent with a benign fibrous process.

Histopathologic findings

Histopathologic examination demonstrated well-circumscribed, collagen-rich lesions composed of a sparsely cellular spindle-cell proliferation (Figure 4). Low- to intermediate-power examination showed a hypocellular fibrous proliferation adjacent to and extending around residual epididymal and tunical structures, without infiltrative growth or atypical features (Figures 5, 6). At higher magnification, the spindle cells had elongated, bland nuclei without cytologic atypia or mitotic activity (Figure 7). Small lymphoid aggregates were present in some areas within the dense fibrocollagenous stroma (Figure 8). Overall, the well-circumscribed, collagen-rich proliferation of bland spindle cells, with no cytologic atypia, mitotic activity, or infiltrative growth and only limited inflammatory change, favored a benign paratesticular fibroma involving the testicular tunics and epididymal tissue. Given the characteristic morphology and absence of concerning histologic features, additional immunohistochemical studies were not considered necessary for diagnosis.

**Figure 4:**
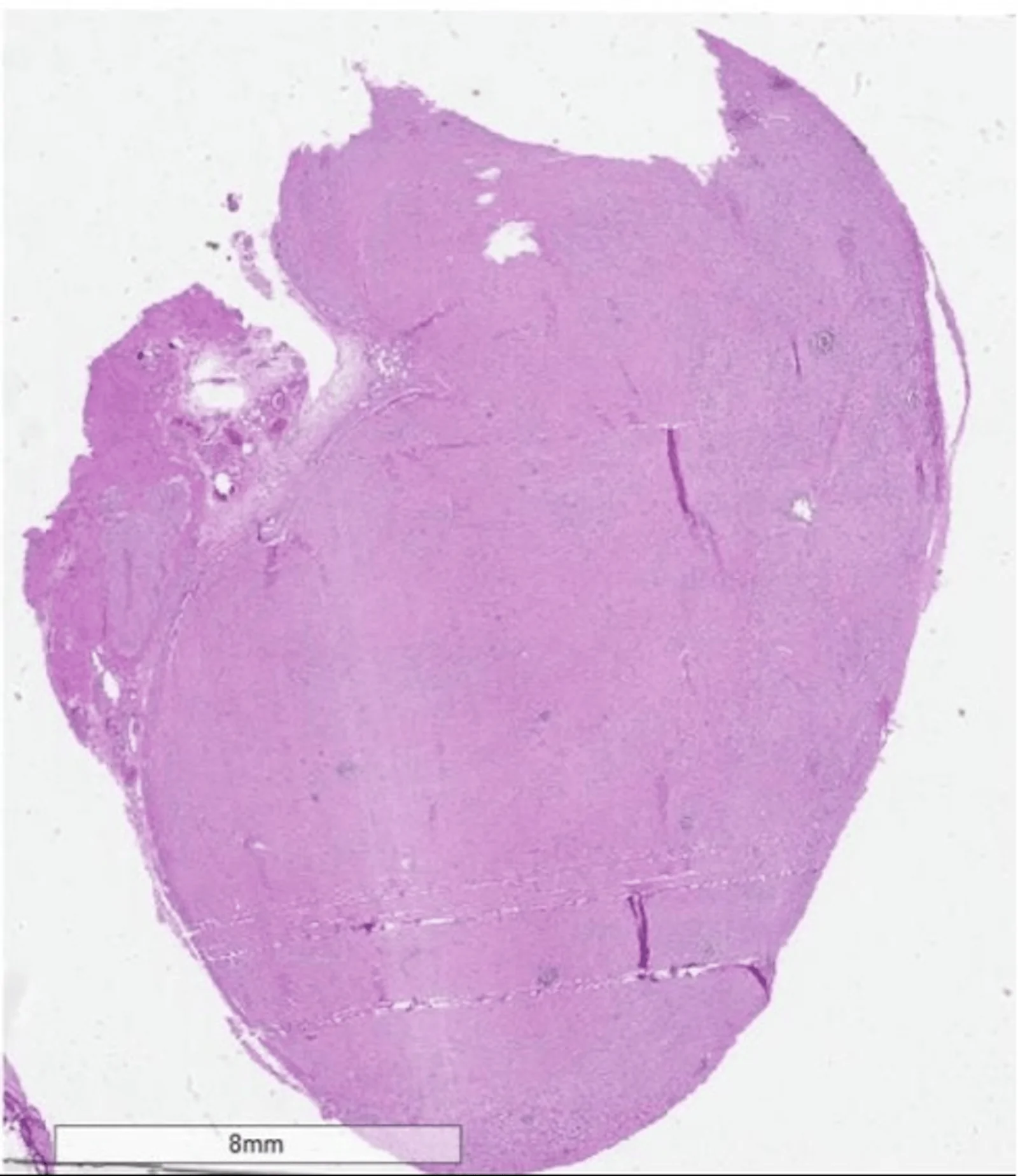
Low-power (H&E) panoramic section demonstrating a well-circumscribed fibrous lesion. The mass appears homogeneous and collagen-rich, without infiltrative borders, consistent with a benign paratesticular fibroma. Scale bar: 8 mm.

**Figure 5:**
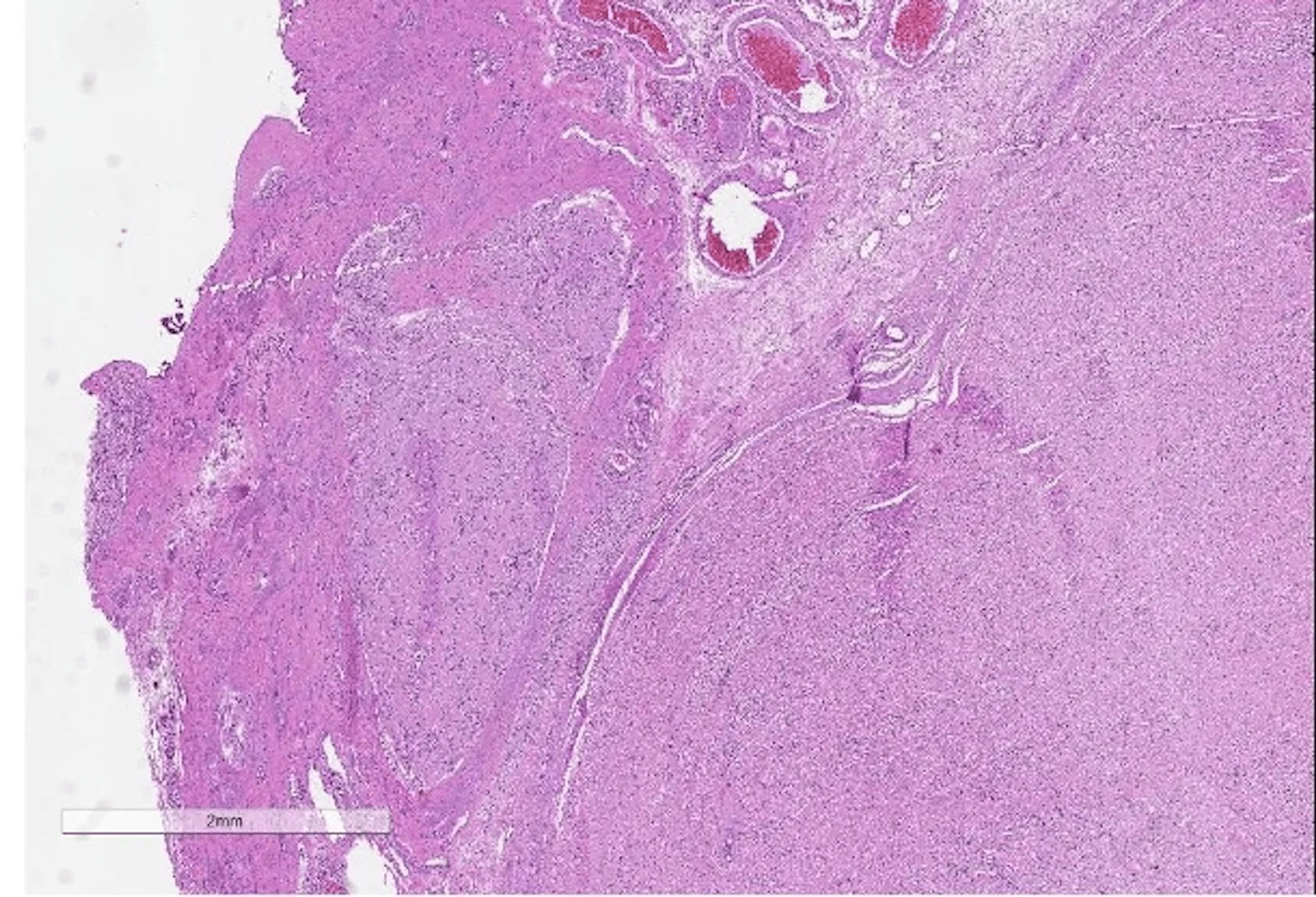
Low- to intermediate-power (H&E) section demonstrating a well-organized fibrous proliferation adjacent to paratesticular structures. The lesion is composed of hypocellular collagenous tissue with scattered spindle cells. Residual ductal/epididymal elements are visible at the upper portion of the field. No infiltrative growth or atypical features are present. Scale bar: 2 mm.

**Figure 6:**
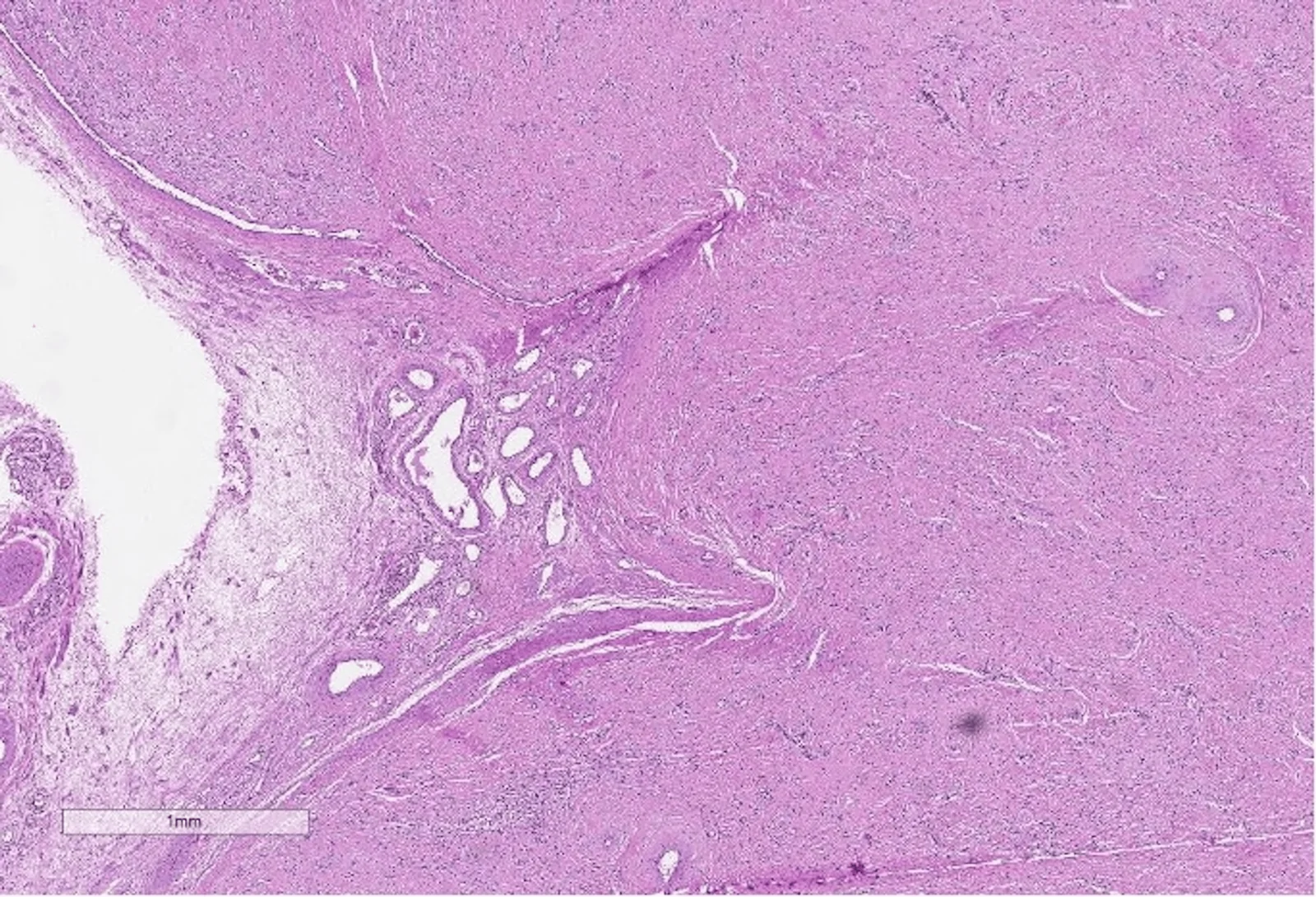
Intermediate-power (H&E) section showing fibrocollagenous proliferation extending around and between residual ductal structures, consistent with involvement of epididymal or tunical tissue. The stroma is sparsely cellular, composed of bland spindle cells within dense collagen. No atypia or infiltrative growth pattern is identified. Scale bar: 1 mm.

**Figure 7:**
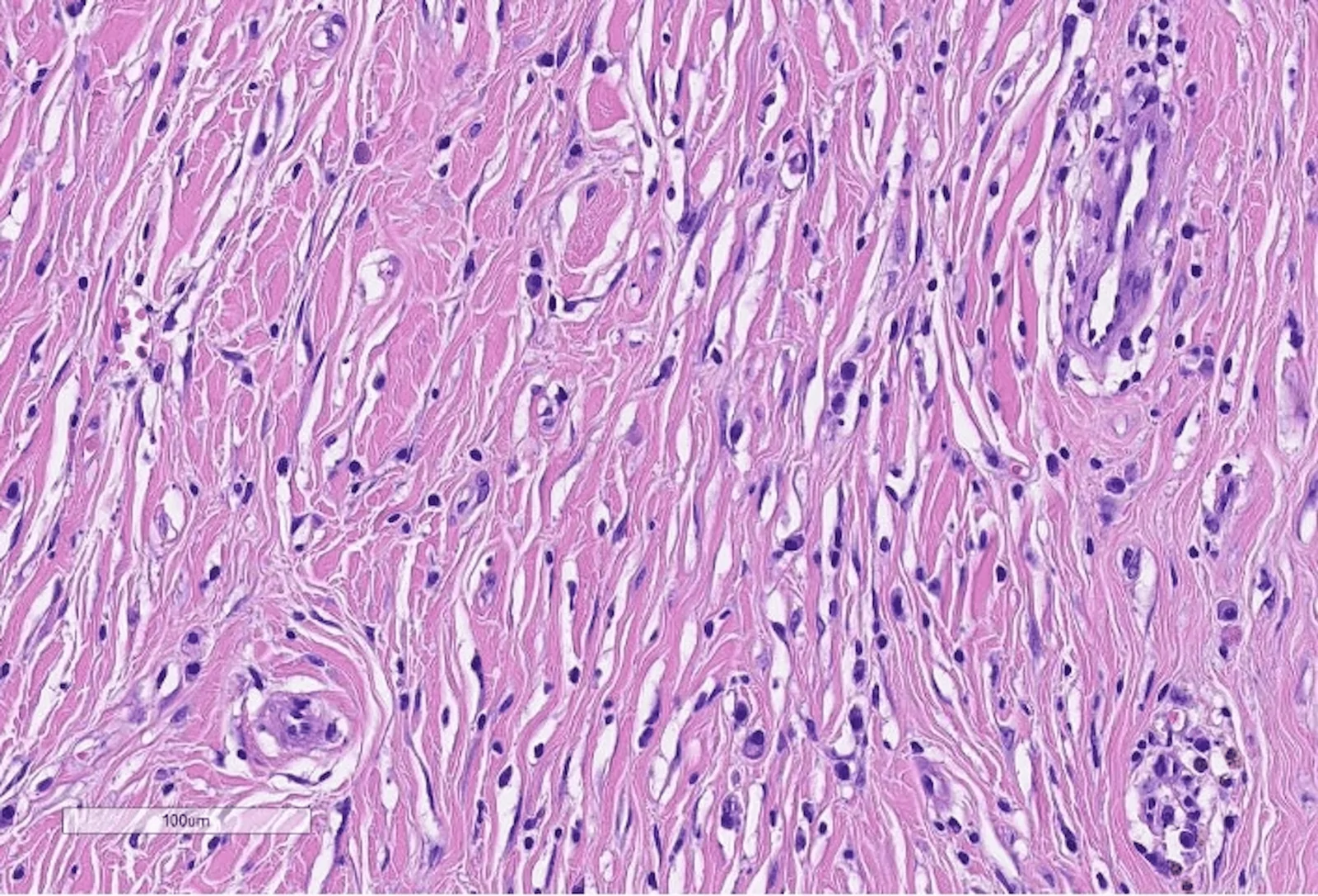
High-power (H&E) photomicrograph showing elongated, bland fibroblasts embedded within dense collagen bundles. The spindle cells exhibit uniform nuclei without atypia or mitotic activity. Occasional small vascular channels and minimal inflammatory elements are present. These features are characteristic of a benign paratesticular fibroma. Scale bar: 100 μm.

**Figure 8:**
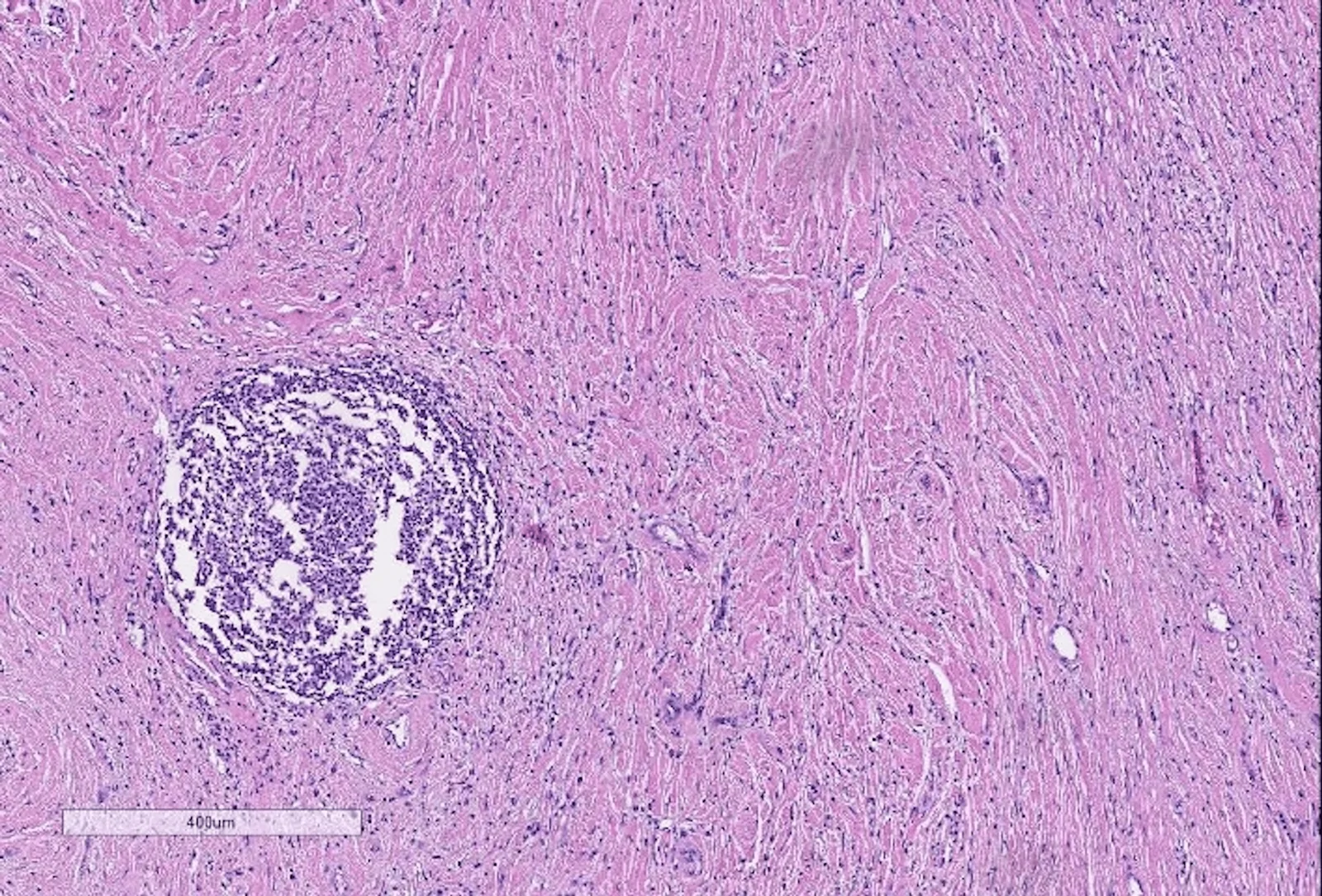
Intermediate-power (H&E) image showing dense fibrocollagenous stroma with scattered bland spindle cells typical of a paratesticular fibroma. A well-formed lymphoid aggregate is present, a feature commonly seen in fibrous pseudotumors of the paratesticular region. No cytologic atypia or mitotic activity is identified. Scale bar: 400 μm.

Follow-up

The postoperative course was uncomplicated. At the six-month follow-up, the patient remained asymptomatic. Repeat scrotal ultrasonography showed no recurrent masses, and the right testis maintained normal size, echotexture, and vascularity.

## Discussion

Paratesticular and testicular fibrous lesions comprise a heterogeneous group, and the terminology used in the literature requires careful interpretation. In particular, true fibromas should not be considered synonymous with fibrous pseudotumors. Jones et al. proposed a classification separating benign neoplastic fibrous lesions, including fibroma of gonadal stromal origin and fibroma of the testicular tunics, from fibrous pseudotumor, which is generally regarded as a reactive fibroinflammatory proliferation [11]. Fibroma of the testicular tunics has likewise been described as a rare benign intrascrotal neoplasm [2]. Although the literature on paratesticular fibrous pseudotumor is relevant to the clinical presentation, imaging appearance, differential diagnosis, and surgical management of fibrous masses, these entities should not be considered pathologically interchangeable. In the present case, the final pathologic interpretation favored fibroma based on the well-circumscribed, collagen-rich proliferation of bland spindle cells, the absence of cytologic atypia or mitotic activity, and the limited inflammatory component. Comparison with fibrous pseudotumor remains relevant because of the substantial clinical and radiologic overlap between these entities.

Fibrous pseudotumor has been reported across a broad age range, although it is most commonly described in young to middle-aged adults. In the 13-case series by Miyamoto et al., patients ranged from 19 to 75 years, with a mean age of 41.9 years, while other reviews have described an even wider age distribution [1,8]. The usual clinical presentation is a slowly developing, firm, frequently painless scrotal mass that may be difficult to distinguish clinically from neoplasia [1,12,13]. The tunica vaginalis is the most frequently involved site in fibrous pseudotumor, whereas the tunica albuginea, epididymis, and spermatic cord are less commonly affected [8]. Epididymal fibrous pseudotumor has nevertheless been described with imaging-pathologic correlation, and lesions involving combinations of the tunica albuginea, tunica vaginalis, and epididymis have also been reported [14,15]. Both solitary and multiple nodular forms occur [6,8,13,15]. Thus, the presence of multiple fibrous nodules with epididymal and paratesticular involvement in the present case falls within the anatomic and morphologic spectrum described for benign fibrous lesions, although multiplicity alone does not distinguish a true fibroma from a reactive fibrous pseudotumor.

Ultrasonography remains the initial imaging examination for a scrotal mass because it can establish whether a lesion is predominantly intratesticular or extratesticular and characterize its solid or cystic nature. However, the sonographic appearance of fibrous lesions is not specific. Reported fibrous pseudotumors may be hypoechoic or approximately isoechoic relative to the testis, may demonstrate posterior acoustic shadowing when collagen or calcification is prominent, and may show variable internal vascularity on Doppler examination [4,8]. Similarly, epididymal fibrous pseudotumor may resemble other solid epididymal or paratesticular masses on ultrasonography [14]. Consequently, although localization to an extratesticular compartment may narrow the differential diagnosis, ultrasonography alone generally cannot definitively distinguish a benign fibrous lesion from a neoplasm [4,7].

MRI is particularly useful when ultrasonographic findings remain indeterminate because the abundant collagen within fibrous lesions produces characteristic tissue-signal properties. Markedly low signal intensity on T2-weighted sequences has repeatedly been described in fibrous pseudotumor and other collagen-rich scrotal lesions [7-9,12,14]. Contrast enhancement is more variable and reflects differences in cellularity, vascularity, inflammation, and collagen content; reported lesions range from relatively limited or heterogeneous enhancement to marked diffuse enhancement [5,8,12]. In the present case, the masses were well circumscribed and hypointense on T2-weighted imaging and compressed the testis without apparent invasion of the testicular parenchyma. The pronounced T2 hypointensity was therefore concordant with the collagen-rich fibrous lesions described in previous reports [8,9,12,14]. The absence of apparent invasion and well-defined margins favored a benign process; however, these findings could not establish the histologic diagnosis or completely exclude malignancy before surgery.

This residual diagnostic uncertainty is clinically relevant because the differential diagnosis of a solid scrotal mass includes both benign and malignant entities. Although many extratesticular masses are benign, malignant paratesticular neoplasms, particularly sarcomas, must remain in the differential diagnosis, while an apparently intratesticular component raises additional concern for a primary testicular neoplasm [4,9]. Imaging is therefore most useful for defining lesion location, extent, relationship to the testicular parenchyma, and tissue composition rather than replacing histopathologic examination. In the present case, histopathologic examination demonstrated dense collagenous tissue containing scattered bland spindle cells without cytologic atypia, mitotic activity, or necrosis, together with small lymphoid aggregates. These findings supported the diagnosis of a benign paratesticular fibroma. Dense hyalinized or collagenous stroma with bland spindle cells is also characteristic of fibrous pseudotumor, although the degree and pattern of inflammation may vary considerably; Miyamoto et al. described plaque-like, inflammatory-sclerotic, and myofibroblastic morphologic patterns [1]. True fibromas of the tunics are likewise composed of cytologically bland spindle or stellate cells within collagenous or variably myxoid stroma. Final classification therefore requires correlation of microscopic architecture, inflammatory features, and anatomic origin, with immunohistochemistry serving as an adjunct when the morphology is diagnostically equivocal [2,11]. In the present case, the histomorphologic findings were considered sufficient for classification, and additional immunohistochemical studies were not considered necessary for establishing the diagnosis.

The inflammatory component of fibrous pseudotumor has also prompted investigation of a possible relationship with IgG4-RD. Bösmüller et al. demonstrated increased numbers of IgG4-positive plasma cells in three paratesticular fibrous pseudotumors and proposed that at least some of these lesions might belong to the spectrum of IgG4-related fibroinflammatory disease [6]. Dieckmann et al. subsequently described two young patients whose paratesticular fibrous pseudotumors showed histologic and immunohistochemical features supporting this association [5]. This relationship should not, however, be extrapolated to all paratesticular fibrous lesions or to true fibromas. In the present case, small lymphoid aggregates were present, but the overall morphology and limited inflammatory component did not suggest an IgG4-related fibroinflammatory process. Therefore, additional IgG4 immunohistochemical evaluation was not considered necessary for establishing the diagnosis.

The principal therapeutic challenge is to obtain a definitive diagnosis while avoiding radical surgery when the lesion can be safely excised. Historically, radical orchiectomy has frequently been performed because these firm fibrous lesions may be difficult to distinguish from malignancy preoperatively [1,16,17]. When the lesion is well circumscribed and technically separable from viable testicular tissue, however, local excision with preservation of the testis has been supported by published experience [10,16,17]. Intraoperative frozen-section examination may further assist surgical decision-making when the diagnosis remains uncertain. In a five-case series, Gordetsky et al. reported that frozen-section analysis contributed to testis-sparing surgery in two patients, while three ultimately required orchiectomy because of concerns regarding testicular viability or residual diagnostic uncertainty [3]. DeCoste et al. similarly reported successful testis-sparing excision aided by intraoperative frozen-section assessment [13]. These observations emphasize that frozen-section analysis is a useful adjunct in appropriately selected cases rather than a guarantee that orchiectomy can always be avoided.

The management and short-term outcome in the present case are consistent with this conservative approach. All visible nodules were excised while the testis was preserved, providing sufficient tissue for definitive histopathologic diagnosis without radical orchiectomy. Published outcomes after complete excision of fibrous pseudotumor have generally been favorable. Zhang et al. reported no recurrence among five patients during a mean follow-up of 26 months, including three patients treated by local excision [16], while the two locally excised cases reported by Dieckmann et al. remained well at 12 years and 18 months of follow-up, respectively [5]. No recurrence was identified in the present patient at six months. Although this duration is insufficient to establish long-term recurrence risk, the short-term outcome is reassuring and is consistent with favorable outcomes reported after complete excision of benign fibrous paratesticular lesions. Given the single-case nature of this report, these findings should be interpreted as illustrative rather than broadly generalizable. Taken together, the clinical course, MRI appearance, benign histopathologic findings, and testis-sparing excision in this case illustrate the potential role of careful preoperative localization and tissue characterization followed by organ-preserving surgery in selected patients when the lesion can be completely removed without compromising oncologic safety.

## Conclusions

This case illustrates the testis-sparing management of a benign paratesticular fibroma in a young patient presenting with a painless scrotal mass. Imaging demonstrated a localized extratesticular process, and intraoperative findings allowed complete excision while preserving the testis. Histopathologic examination confirmed a benign fibroma, and no recurrence was identified during the six-month follow-up period. Although longer follow-up is needed to assess long-term recurrence, this case illustrates that organ-preserving surgery may be considered in carefully selected patients when the lesion can be completely excised without compromising oncologic safety.
